# Chronotype in alpha-tACS: Preliminary evidence hints at sleep quality modulation of aftereffects in evening types in the morning

**DOI:** 10.1016/j.nbscr.2025.100136

**Published:** 2025-11-07

**Authors:** Peppi Schulz, Heiko I. Stecher, Christoph S. Herrmann

**Affiliations:** a Experimental Psychology Lab, Department of Psychology, Carl-von-Ossietzky Universität Oldenburg, Ammerländer Heerstr. 114-118, Oldenburg, 26111, Lower Saxony, Germany; b European Medical School, Cluster for Excellence “Hearing for All”, Research Center Neurosensory Science, Carl-von-Ossietzky-Str. 9-11, Oldenburg, 26129, Lower Saxony, Germany; c Research Center Neurosensory Science, Carl-von-Ossietzky Universität Oldenburg, Carl-von-Ossietzky-Str. 9-11, Oldenburg, 26129, Lower Saxony, Germany

**Keywords:** Transcranial alternating current stimulation, EEG, Chronotype, Sleep quality, Aftereffects, Sustained attention, Alpha power

## Abstract

Transcranial alternating current stimulation (tACS) is a promising tool for research on oscillatory brain activity, yet both behavioral and electrophysiological outcome measures show high variability across studies. One source for this variability might be chronotype and an incidental mismatch between chronotype and the time of the measurement.

14 evening type and 14 morning type participants performed a sustained attention task — once at their chronotypically optimal and once at a non-optimal time of day. TACS was applied for 20 min at the individual alpha frequency over two electrodes located at Cz and Oz. EEG was recorded for 10 min prior to and after stimulation. Sleep timing and quality were assessed with a sleep questionnaire. While planned analyses failed to find effects of stimulation and session timing on alpha power, exploratory analyses revealed that below average sleep quality in evening types in the morning was associated with no changes or unexpected decreases in alpha power after stimulation. Effects of sleep quality were present in the morning for evening types, but neither in the evening session nor in morning types. It is suggested that this effect of sleep quality reflects increased sleepiness, which could impede expected aftereffects of tACS. It is likely that effects of sleepiness might be especially relevant when people are stimulated at a chronotypically non-optimal time. Due to the exploratory nature of these sleep effects and their presence in only a small subgroup leading to low power and confidence, future systematic sham-controlled studies are needed to clarify the relationship between sleep, time of day and chronotype in α-tACS proposed here.

## Highlights

• No direct effect of chronotype or circadian phase on α-tACS outcome.
• Sleep quality of evening types before the morning session correlates with post-stimulation α-power.
• High variability in electroencephalographic outcomes stress the need for control of individual sources of variability such as sleep quality and sleepiness.

## 1 Introduction

Virtually all lifeforms follow some sort of daily rhythm (Vitaterna et al., 2001). In humans, circadian modulation of simple physiological processes, (e.g. regulating core body temperature Coiffard et al., 2021 ) and hormonal levels (Cajochen et al., 2003, Mohd Azmi et al., 2021), as well as the effects of circadian disruptions due to mismatches between a person’s chronotype and their daily schedule (Meyer et al., 2022) have been well described. In recent years, research into circadian modulation of human functioning has extended into cognition and neuroplasticity (Xu et al., 2021). It has been shown that the circadian rhythm modulates cortical excitability (Tamm et al., 2009), as well as neuroplastic processes (Snider et al., 2018), of which some are underlying lasting effects of non-invasive brain stimulation (NIBS) methods, such as transcranial alternating current stimulation (tACS) (Vogeti et al., 2022).

TACS shows great potential for research into the causal role of oscillatory activity (Herrmann et al., 2016) and as a therapeutic tool for disorders associated with altered brain oscillations (Elyamany et al., 2021). However, tACS studies face high variability. Some studies found strong, lasting effects (Kasten et al., 2016, Zähle et al., 2010), while others failed to replicate these findings despite employing seemingly similar setups (Fekete et al., 2018, Veniero et al., 2017, Clayton et al., 2018). It has been suggested that inter-subject variability in stimulation receptivity might explain parts of these seemingly conflicting results (Krause and Cohen Kadosh, 2014). One such factor might be the (mis-)match between a participant’s chronotype and the time of day during stimulation and resulting disruptions of neuroplastic processes at chronotypically non-optimal times (Sale et al., 2010, Salehinejad et al., 2021, Martínez-Pérez et al., 2022).

Mismatches between the time of measurement and a person’s chronotype have been shown to reduce performance in vigilance tasks, with evening types showing especially large performance decreases at non-optimal times Facer-Childs et al. (2018). Similar effects have also been shown in memory (Schmidt et al., 2007), attention and inhibition (Taillard et al., 2021), and academic performance (Sabaoui et al., 2022). These findings fit the model of the synchrony effect, which describes enhanced performance when assessment time and peak circadian arousal align and is especially prominent in people with strong chronotypes in tasks involving high cognitive effort and control (May et al., 2023).

Effects of circadian modulation and chronotype can also be observed in the EEG. In a forced desynchrony design, disentangling effects of endogenous circadian phase and sleep–wake patterns, Cajochen et al. (2002) showed that posterior alpha was lowest at circadian phases representing endogenous times of activity and highest at circadian phases representing endogenous times of sleep, irrespective of the actual prolonged sleep–wake pattern. Taillard et al. (2003) found a slower and later increase in peak theta-alpha (6 Hz to 9 Hz) frequency power in evening types compared to morning types in response to a 36 h constant awake routine. The effects of chronotype on the EEG during the normal day are less well studied. Some studies suggest interactions between chronotype and time of day, with enhanced oscillatory activity at optimal times (Venkat et al., 2020), while others fail to find such differences (Salehinejad et al., 2021). More research is clearly needed to correctly evaluate this relationship and disentangle it from typical time on task effects (Pershin et al., 2023).

The effects of the alpha rhythm are assumed to be mostly inhibitory by suppressing the activity of task irrelevant areas and therefor allowing the brain to concentrate on the task at hand (Klimesch et al., 2007).

While tasks requiring flexible shifts of attention between several locations often show low posterior alpha-power (Bauer et al., 2014, Foxe and Snyder, 2011), sustained attention tasks show strong correlations of better performance with enhanced posterior alpha power (Clayton et al., 2019), solidifying the role of alpha-oscillations for focusing attention and suppressing distractors (Bonnefond and Jensen, 2012).

Neuroplasticity describes “the ability of the nervous system to change its activity in response to intrinsic or extrinsic stimuli by reorganizing its structure, functions, or connections” (Mateos-Aparicio and Rodríguez-Moreno, 2019). One key concept underlying neuroplasticity is spike-timing-dependent plasticity (STDP) (Song et al., 2000). In classic (“Hebbian”) accounts of STDP, long-term potentiation (LTP) occurs when presynaptic spikes precede postsynaptic spikes and vice versa for LTD (Feldman, 2012). It has been suggested that tACS can influence this spike timing, leading to STDP-like processes, reflected in lasting power increases at the stimulation frequency (Vogeti et al., 2022). Various forms of STDP depend fully or partly on the N-methyl-D-aspartate receptor (NMDAR) (Andrade-Talavera et al., 2023). Wischnewski et al. (2019) found that giving participants an NMDAR blocker (dextromethorphan) lead to the absence of aftereffects of beta-tACS over the motor cortex, which were present in the placebo condition. NMDAR activation depends on glycine and L-glutamate binding (Zhu et al., 2016). It has been shown, that these neurotransmitters are modulated by and involved in circadian rhythms (Castañeda et al., 2004, Frenkel et al., 2017). Furthermore, for the induction of LTP- and LTD-like changes, a reduction of GABAergic activity seems to be required (Liebetanz et al., 2002), which is also associated with changes during the circadian rhythm (Ono et al., 2018).

The present study was designed to investigate the effects of chronotype on tACS induced aftereffects in the alpha band. Specifically, its aim was to clarify some of the large inter-individual variability commonly observed in tACS studies by taking the potential role of a chronotype and time-of-day interaction into account. Considering previously reported time-of-day-dependent, chronotype-specific effects in the EEG and in other NIBS protocols, the present study examined whether similar effects appear in tACS. Furthermore, the effects of sleep time and quality were considered to potentially disentangle the effects of sleep from those of chronotype and time-of-day congruency. This study was focused on chronotype effects as a potential factor that could lead to differences between active tACS conditions, we opted to omit a sham group comparison, both to limit the scope of the study and because occipito-parietal tACS has been shown to increase alpha-activity compared to sham in principle before (see Veniero et al. 2015). To retain comparability with previous, sham-controlled studies, we employed a previously established stimulation and recording setup (Kasten et al., 2016) and used a similar visual vigilance task.

Assuming neuronal plasticity to have a strong causal role in the occurrence of tACS aftereffects, it seems likely that a chronotype and time-of-day interaction previously reported in endogenous and NIBS induced plasticity can also be seen in terms of the occurrence and strength of tACS aftereffects. Thus we hypothesize that:

1. Occipito-parietal tACS at individual alpha frequency applied at a chronotypically optimal time of day induces significantly stronger aftereffects compared to a non-optimal time.
2. Alpha activity prior to stimulation during a sustained attention task is significantly stronger at the optimal time of day as determined by higher resting state frequency and power measured at parietal EEG locations, reflecting enhanced performance due to the synchrony effect.
3. Both effects are not sufficiently described by only taking sleep time and quality into account.

## 2 Methods

### 2.1 Participants

28 young adult volunteers (*M*_age_ = 25.22, *SD* = 3.24), who were either identified as evening- (10 moderate and 4 definite) or morning-types (4 moderate and 10 definite), were recruited from the student population of the Carl-von-Ossietzky Universität Oldenburg. 60.7% identified as cisgender men, 39.3% as cisgender women. The gender distribution in both subgroups was not equal (28.6% cisgender women in the evening type group but 50% cisgender women in the morning type group), reflecting that women until the age of 30 are more likely to be morning types than men of the same age (Duarte et al., 2014). Chronotype was determined with the MEQ in a prior screening session, for which 45 participants were recruited in total. For these screening sessions, recruitment ads targeting people who would consider themselves to be either evening or morning people were posted on the university’s e-learning platform. Inclusion criteria were age between 18 and 36 and an MEQ score below 42 for the evening-type subgroup or above 58 for the morning-type subgroup, indicating at least a moderate chronotype. Exclusion criteria were the presence of magnetizable metal in the skull or brain, cochlea-implants, neurostimulators, a history of epilepsy for the participant and their close family, pregnancy, taking medication affecting the central nervous system and presence or history of neurological or psychiatric disease. These exclusion criteria follow standard recommendations for tACS studies (Antal et al., 2017).

Based on a questionnaire about the preferential hand use for daily activities, adapted from the Edinburgh handedness inventory (Oldfield, 1971), 21 participants were identified as right handed, 2 as left-handed and 5 as ambidextrous. Alcohol consumption was controlled in a questionnaire before each session, no participant reported alcohol consumption on the same day, 3 reported light (1 drink) consumption on the day before. Most participants consumed no caffeine prior to the measurements, 3 reported very light caffeine intake (less than half a cup of coffee/one small tea), 8 reported light caffeine usage (1 cup of coffee/tea or less) and 4 reported moderate caffeine consumption. All participants had normal or corrected-to-normal vision.

Participants were remunerated with 12€ per hour for their participation. Participants were informed about the experimental procedure prior to the start and debriefed, including an explanation of expected effects, at the end of the last session. All gave their written consent for participation and data analysis. The study was approved by the local ethics committee and was in accordance with the declaration of Helsinki (World Medical Association, 2024). The study was preregistered on Open Science Framework prior to data collection (https://osf.io/qw286).

### 2.2 Power

An a priori power analysis using GPower 3.1 (Faul et al., 2007) was conducted to determine the ideal sample size for a robust repeated measures ANOVA. Assuming effect sizes typically found in other tACS studies focusing on alpha power change which have been reported as between f=0.47 (Kim et al., 2020) and f=0.65 (Kasten et al., 2016) resulted in an ideal sample size between 38 and 22, given an alpha level of .05 and a desired power level of .80, using the recommended but non-default conservative effect size estimation (Cohen, 1988). Given the actual sample size of 28, a post-hoc analysis revealed that a true effect size of f=0.55 or larger would be necessary to achieve the desired minimum power level of 80%. To achieve 95% power for a more reliable estimate, an effect size of at least f=0.71 would have been needed. This suggests that the achieved sample size would be sufficient, albeit slightly underpowered, to detect a similarly large effect. However, as effect sizes above f=0.40 typically reflect relatively large effects (Cohen, 1988), it is possible that, if we overestimated the true effect size, our sample size and the associated power could be too low to reliably detect a smaller effect.

### 2.3 Experimental design

The present study consisted of three sessions. During a first screening session, the chronotype of potential participants was evaluated using either the original english version of the Morningness-Eveningness Questionnaire (MEQ) (Horne and Ostberg, 1976), or the equivalent German D-MEQ (Griefahn et al., 2001). Participants were classified in one of five groups according to the original German norms (Griefahn et al., 2001) and all eligible non-intermediate types were then invited for two tACS sessions in the following days. Suitable candidates were pseudo-randomly assigned to either receive tACS at a time congruent with their chronotype (16:00 for evening and 09:00 for morning types) or incongruent with their chronotype (09:00 for evening and 16:00 for morning types) in the first session. The second session was scheduled at least 24 h later at the respective other time. Other than the differing starting times, both tACS-sessions followed the same procedure.

**Fig. 1:**
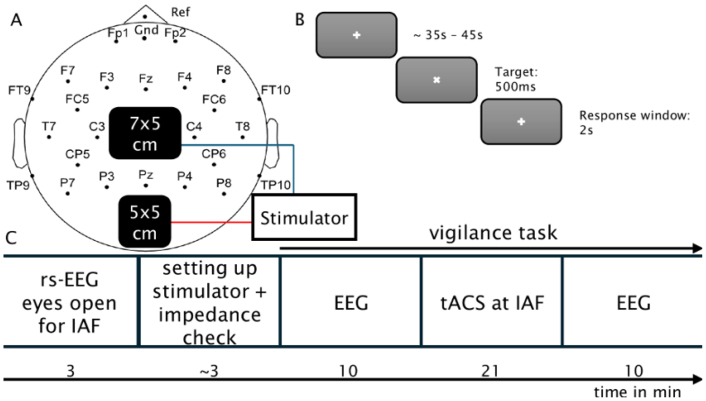
**Experimental Procedure(A)** EEG and tACS Electrode Setup: Stimulation electrodes were centered on Cz and Oz in the international 10-10 system. 24 EEG electrodes were recorded as marked and referenced to a central reference on the nose. Ground was at FPz. **(B)** Visual Vigilance Task: Participants were asked to fixate on a small white cross in the center of a gray screen. After a random time period between 35 s and 45 s, the fix-cross was rotated by 45° for 500 ms. Participants had 2 s to respond to the rotation by pressing a button using their preferred index finger. **(C)** Time Course of the Experiment: First, 3 min of resting state EEG with participants keeping their eyes open and fixating on a stationary fix-cross were acquired. This data was used to determine IAF, which served as the stimulation frequency. After successful determination of a clear alpha peak, the room was entered again to turn on and attach the stimulator and perform a last impedance check. Then, participants performed a visual vigilance task for 41 min. For the first 10 min, EEG was measured. Then, 21 min of tACS at IAF, 2 mA peak to peak, including 30 s of linear fade-in and fade-out followed. For the last 10 min, EEG was measured again.

Each tACS session started with a short briefing. Consumption of caffeine, alcohol and nicotine was recorded with a self-report questionnaire. Then, participants completed the “Schlaffragebogen-A” (Görtelmeyer, 2011) to assess sleep time and quality in the previous night against a population norm. Afterwards, both tACS and EEG electrodes were applied. Participants were then seated in a dimly lit, electro-magnetically shielded room in front of a 24 inch computer screen, located behind a window outside the room. Live EEG was shown to the participants to visualize the effects of excessive movements, blinking and yawning. Participants performed a visual vigilance task, which is described in Fig. 1 C. All participants received active stimulation in both sessions. After each session, a short debriefing took place to monitor potential side-effects. Average preparation time before start of recording was 146.5 min (max: 190, min: 94, std: 21.6).

### 2.4 Electroencephalography

The EEG was recorded at a sampling rate of 1 kHz from 24 active sintered Ag-AgCl electrodes, fitted in an elastic cap (EASYCAP GmbH, Herrsching, Germany), connected to an actiCHamp amplifier (Brain Products GmbH, Gilching, Germany). The reference electrode was placed on the tip of the nose and the ground electrode was placed on FPz. Impedances were kept below 20 kΩ. The signal was recorded using the PyCorder 1.0.9. software. The recording channels were placed according to the 10/10-system, omitting sites placed under or directly neighboring the tACS-electrodes (see Fig. 1 A).

### 2.5 Electrical stimulation

Stimulation was performed using a battery powered neuroConn DC-

**Fig. 2:**
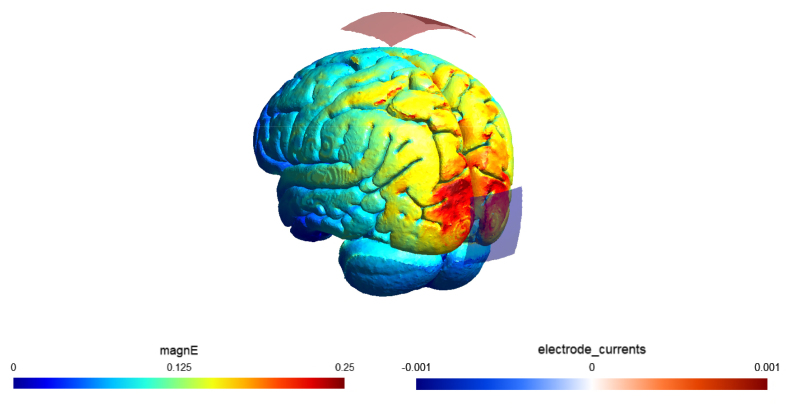
SIMNIBS-based current simulation of the electrical field induced by tACS.

STIMULATOR PLUS (neuroConn GmbH, Illmenau, Germany). Electrode montage and stimulation settings followed a standard approach for occipito-parietal alpha stimulation to facilitate comparability with previous studies: one 5 × 7 cm surface conductive-rubber electrode at Cz and one 5 × 5 cm electrode at Oz in the international 10-10 system, a stimulation intensity of 2 mA peak to peak and stimulation at peak IAF (Vogeti et al., 2022). A 21 min sinusoidal stimulation signal with a 30 s fade in and fade out period was generated in MATLAB R2022b (The MathWorks Inc, Natick, Massachussets) and sent via a digital-to-analog converter (DAQ NI USB 6229, National Instruments, Austin, TX, United States) to control the stimulator remotely. The rubber electrodes were attached to the participants’ head with Ten20 Paste (D.O. Weaver, Aurora, CO, United States). Stimulation was only performed if impedances were below 10 kΩ. This check was performed twice, once immediately after applying the tACS electrodes and once after applying the EEG electrodes, just before the start of the actual measurement. A simulation of the induced electrical field is presented in Fig. 2.

### 2.6 Data analysis

Data was analyzed using the Fieldtrip toolbox (Oostenveld et al., 2011) in MATLAB 2022b (The MathWorks, Inc., Natick, MA, United States)

#### 2.6.1 IAF

To estimate IAF during the experiment, resting state EEG data was loaded and down-sampled to 250 Hz to reduce processing time. Data was de-meaned and de-trended first. Faulty channels were visually identified and removed. An independent component analysis (ICA) was performed to remove eye blink artifacts. For this, data was filtered between 2 Hz and 30 Hz and split into 2 s epochs. Epochs with unusually high peaks (i.e. larger than 300 µV) were automatically removed. ICA components reflecting blinks and eye movement artifacts were manually selected based on a pre-determined, structured approach. Component rejection was applied to the unfiltered, non-epoched data. Next, the data was epoched to the same length again and filtered between 1 Hz and 48 Hz. Epochs with peaks larger than 150 µV were automatically removed. A Fast-Fourier Transformation was used to calculate frequency power between 2 Hz and 40 Hz. Data was zeropadded to 10 s and tapered using hanning windows. The 1/f-component was removed using the FOOOF (Donoghue et al., 2020) algorithm as implemented in the FieldTrip toolbox with default settings. IAF was determined as the highest amplitude peak between 7 Hz and 13 Hz in channel Pz using the Matlab *findpeaks()* function. Channels P3 and P4 were additionally checked to see if results align. In case of inconclusive results, the IAF recording was repeated.

#### 2.6.2 EEG data

All offline analyses were performed on non-resampled data. Each recording was split into a pre-stim, stim and post-stim block. Only the pre-stim and post-stim blocks, appended to one single EEG file, were analyzed further, due to the large tACS artifact masking underlying EEG activity during stimulation.

Faulty channels were visually identified and removed. The combined data was then highpass-filtered at 2 Hz and lowpass-filtered at 40 Hz with 6th Order Butterworth Filters, following recommendations to employ strong high-pass filters to improve the quality and accuracy of ICA components for EOG artifact removal (Winkler et al., 2015, Dimigen, 2020). Then, an ICA was conducted on the filtered data using the extended “runICA” algorithm as implemented in FieldTrip. The time-course, topographies and averaged spectra of the independent components were visually analyzed and components which clearly represented eye movements, heartbeat or blinks were marked. The obtained un-mixing matrix was then applied to the unfiltered data and the marked components were rejected.

Subsequently, the data was highpass-filtered at 1 Hz and lowpass-filterered at 40 Hz with 6th-order Butterworth filters. Data was then de-trended, de-meaned and epoched into 4 s windows (50% overlap). Windows containing peaks larger than 125 µV were automatically discarded. Each window was multiplied with a Hanning taper and zero-padded to 8 s. An FFT was calculated for each window. The resulting powerspectra were then averaged per block. A correction for the 1/f component was performed using the FOOOF algorithm. As the default FieldTrip settings for the FOOOF algorithm led to a sub-optimal fit of the estimate of aperiodic activity, a toolbox for automatic model selection to improve model fit without the reliance on arbitrary decisions for the settings of the hyperparameters was used (Wilson et al., 2024). The aperiodic estimate was then subtracted from the original powerspectrum in linear rather than log–log space, which deviates from Donoghue et al. (2020) but follows Kasten et al. (2024). Subsequently, the 1/f-corrected powerspectra were averaged over channels Pz, P3, P4, P7 and P8. The peak between 7 Hz and 13 Hz in this average powerspectrum was extracted using the MATLAB *findpeaks()* function. IAF was defined as this peak’s frequency and power.

### 2.7 Statistical analysis

Statistical Analyses were conducted using Matlab R2022b and IBM SPSS Version 29.0. The planned main model to test for the effects of chronotype on tACS efficacy was a 2 by 2 repeated measures ANOVA with absolute power at peak IAF as the dependent variable and with the within-subject factors condition (“congruent” vs. “incongruent”) and block (“pre” vs. “post”). For the evaluation of potential differences in reaction times and accuracy in the vigilance task, two 2 by 3 repeated measures ANOVAs were planned, with the factors (“morning” vs. “evening”) and block (“pre” vs. “stim” vs. “post”). Differences between conditions in the sleep questionnaire and the reports of adverse effects were assessed by two-sided t-tests. If model assumptions were violated, either non-parametric alternatives were used or data was normalized. If not otherwise stated, all significance tests were two-sided and alpha was conventionally set at .050. When applicable, Bonferroni corrections were used for multiple comparisons.

## 3 Results

### 3.1 Adverse effects

Most participants reported few and mostly weak adverse effects. The most commonly reported side effects included fatigue (57.14%) and difficulty concentrating (67.86%). The majority of participants reported the duration of the experiment and the relatively simple task as the most likely cause for these two effects. Relatively few participants reported experiences of pain (14.29%), with only two participants rating this higher than 2. The most commonly reported side effect with a clear relation to the stimulation was a tingling (50%) or itching sensation (21.43%). Several of those who reported itching or tingling sensations in the adverse effect questionnaire wrote that this sensation was the strongest during the first 30 to 60 s of stimulation and that afterwards the sensation faded out. Two participants reported a short sensation of phosphenes in the beginning. Another participant reported back pain due to prolonged time of sitting. For an overview of adverse effects split per session see Table 1. In the chronotype congruent session, significantly more participants (N = 5) reported mild headaches, compared to the incongruent session (N = 2). There was no significant difference in other reported adverse effects between conditions as assessed by two-tailed paired t-tests.

**Table 1:** Average Ratings of Adverse Effects Split per Condition. Ratings based on a standard questionnaire for tES adverse effects (Brunoni et al., 2011). For the intensity rating the scale goes from 1 (“none”) to 4 (“strong”). In case a participant rated any adverse effect higher than 1 they were asked to rate whether they believe the stimulation to be the cause of this effect on a similar scale from 1 (“no”) to 4 (“definitely”). Other reported side effects were backpain (*N* = 1), feeling cold (*N* = 1) and experiencing phosphenes (*N* = 2).

	Intensity				caused by tACS?			
	Congruent		Incongruent		Congruent		Incongruent	
Adverse Effect	*M*	*SD*	*M*	*SD*	*M*	*SD*	*M*	*SD*
Headache	1.21	0.49	1.07	0.67	3.00	0.89	2.50	0.50
Neck Pain	1.29	0.65	1.29	0.56	2.00	0.89	1.57	0.73
Scalp Pain	1.25	0.69	1.21	0.45	4.00	0.00	2.40	1.01
Tingling	1.68	0.76	1.61	0.34	3.14	0.83	3.00	0.58
Itching	1.36	0.77	1.43	0.23	2.67	0.47	3.29	0.70
Heating Up	1.18	0.47	1.14	0.12	4.00	0.00	4.00	0.00
Skin Redness	1.04	0.19	1.07	0.01	4.00	0.00	2.00	0.00
Fatigue	1.96	0.98	2.25	0.00	1.75	0.83	1.59	0.77
Difficulty Concentrating	2.04	0.87	2.18	0.00	1.83	1.01	1.61	0.83
Mood Swings	1.29	0.52	1.18	0.00	1.33	0.75	1.20	0.40
Other	1.11	0.42	1.11	0.42	2.00	1.00	3.50	0.50

### 3.2 Behavioral effects

#### 3.2.1 Vigilance task

Average accuracy ($\frac{hits}{hits+misses}$) across conditions was 95.68% (SD = 9.26%), suggesting that the vigilance task at hand was rather easy (hit rate of 100% in 29 out of 58 measurements). One participant in the morning types group showed a hit-rate of 36.67%, which suggest non-compliance with the task and was therefor excluded from further analyses, reducing the total sample size to 27.

As neither raw accuracy nor raw reaction times were normally distributed, both were normalized by dividing the post-stimulation average by the pre-stimulation one. A student’s t-test (paired two-sample) comparing normalized accuracy after stimulation between the incongruent condition (*M* = 1.060, *SD* = 0.151, 95% CI [0.640, 1.478]) and the congruent condition(*M* = 1.031, *SD* = 0.159, 95% CI [0.623, 1.439]) showed non-significant, minimal performance differences after stimulation (*t*_26_ = 1.58, *p* = .13, 95% CI [−0.0085, 0.0641]). To test for baseline differences in pre-stimulation accuracy, a Wilcoxon signed rank test was performed but found no significant differences (*W* = 10, *z* = −1.51, *p* = .13).

A student’s t-test (paired two-sample) comparing normalized reaction times after stimulation between the incongruent condition (*M* = 1.010, *SD* = 0.148, 95% CI [0.609, 1.406]) and the congruent condition(*M* = 0.996, *SD* = 0.154, 95% CI [0.602, 1.389]) showed no significant performance differences after stimulation (*t*_26_ = −0.30, *p* = .77, 95% CI [−0.0707, 0.0946]). To test for baseline differences in pre-stimulation reaction time, a Wilcoxon signed rank test was performed but found no significant differences (*W* = 211, *z* = 0.53, *p* = .60).

#### 3.2.2 Sleep questionnaire

The results of the “Schlaffragebogen-A” showed considerable differences both between conditions and participants. Normalized sleep time was significantly lower in the morning compared to the evening (*t*_26_ = −3.56, *p* = .002, 95% CI [−1.40, −0.37], *d*_av_ = −0.68 (medium effect)). On average, participants slept 7.2 h (*SD* = 1.32) before the morning and 8.3 (*SD* = 1.16) h before the evening session. Three participants, all evening types, reported sleep times of 5 h or less (z_population_ < −1.96). Interestingly, both chronotypes reported sleep times of about 8.3 h before the evening session. However, before the morning session, evening types reported an average of 6.9 h of sleep, while morning types reported 7.5 h of sleep.

**Fig. 3:**
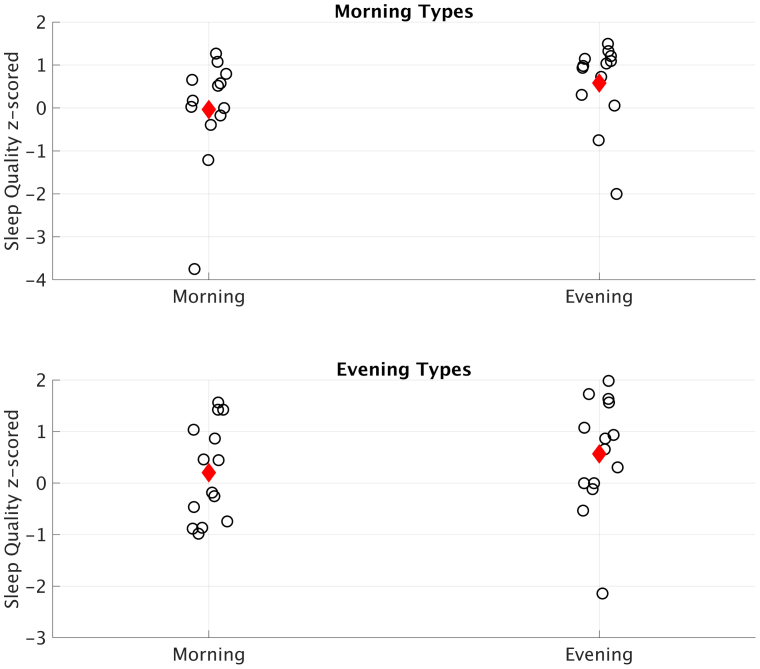
Normalized sleep quality scores of each individual participant split per time of day and chronotype. Black circles reflect individual scores, red diamond reflects the group mean.

Similarly to sleep time, normalized sleep quality was lower in the morning (*M* = 0.01, *SD* = 1.11) compared to the evening (*M* = 0.57, *SD* = 1.03), although this difference was not significant (*t*_26_ = −1.82, *p* = .08, 95% CI [−1.03, 0.06], *d*_av_ = −0.35 (small effect)). However, sleep time showed no correlation with sleep quality (*r*_25_ = 0.04, p = .78). While there was no statistical difference in a two-sample t-test on average sleep quality between chronotypes (*t*_52_ = 0.38, *p* = .71, 95% CI [−0.49, 0.71]), visual analysis of individual sleep quality scores split per chronotype and time of day showed that more evening types reported below average sleep quality, especially in the morning session, whereas morning types reported average or better sleep quality with only two exceptions (see Fig. 3).

### 3.3 Electrophysiological effects

Visual inspection of absolute power at peak IAF in the pre and post block revealed a severe violation of the normality assumption. IAF power followed a right skewed distribution with most observations being very low in power (between 0.3 µV and 1 µV and a few higher power observations. Shapiro–Wilk tests supported this observation (all *p* < .05). Instead of the planned simple comparison of absolute power between pre- and post-stimulation measurements as single blocks, the pre-stimulation block was split into two blocks of 5 min each. Power in the second pre-stimulation and the post-stimulation block was then normalized to the first block before the stimulation period. This normalization was based on a similar approach used in previous research focusing on tACS aftereffects, while still including a time factor allowing a distinction between effects before and after stimulation (Kasten and Herrmann, 2017, Stecher et al., 2017). Keeping this time factor by splitting the pre-stimulation block in half was necessary, as we had no sham group, which would have allowed us to investigate stimulation specific effects with normalized post-stimulation data only.

**Fig. 4:**
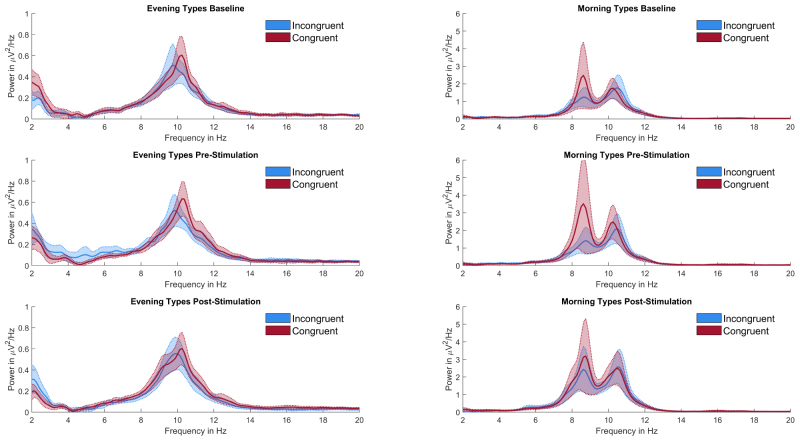
Power spectra split per chronotype and session type. Power is averaged over all parietal electrodes. Left hand side depicts mean power of all evening types, the right part shows the mean power of all morning types. Note the different scaling for the y-axes to increase legibility, as evening types incidentally showed lower power average power values than morning types across blocks and conditions. Furthermore, in the morning types group the mean power spectrum showed two distinct peaks in the alpha range. This seems to be driven by a small subgroup of 7 Morning (5 women) types who showed such double peaks at above average non-normalized amplitudes. This might either reflect two distinct overlapping rhythms, often coined lower and upper alpha (Klimesch et al., 2006) or a phenomenon called split-alpha (Robinson et al., 2003), which might be more common in women (Chiang et al., 2011), but its true origin is under debate (cf. Olejarczyk et al., 2017, Zalewska, 2020). Due to the low number of double peak observations in our study and the unclarity about the nature of these peaks, we opted to analyze only the clearest alpha peak, i.e. each individual’s highest alpha peak in each block averaged over all parietal electrodes.

**Fig. 5:**
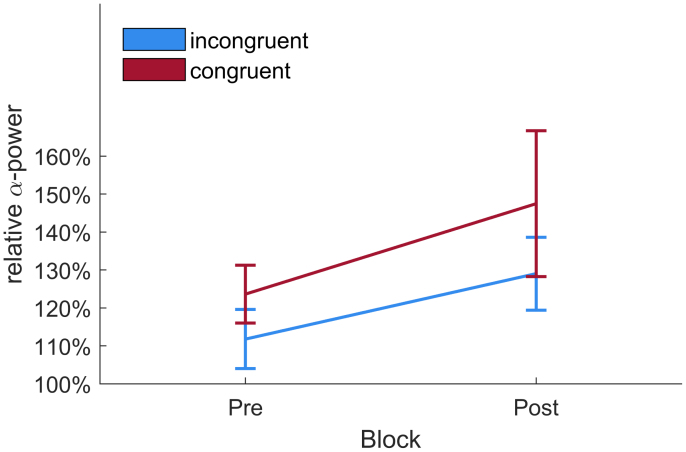
Average normalized power at peak IAF from pre-stimulation to post-stimulation and session type for all valid participants. Power is normalized to first 5 min of the 10 min pre-stimulation block.

**Fig. 6:**
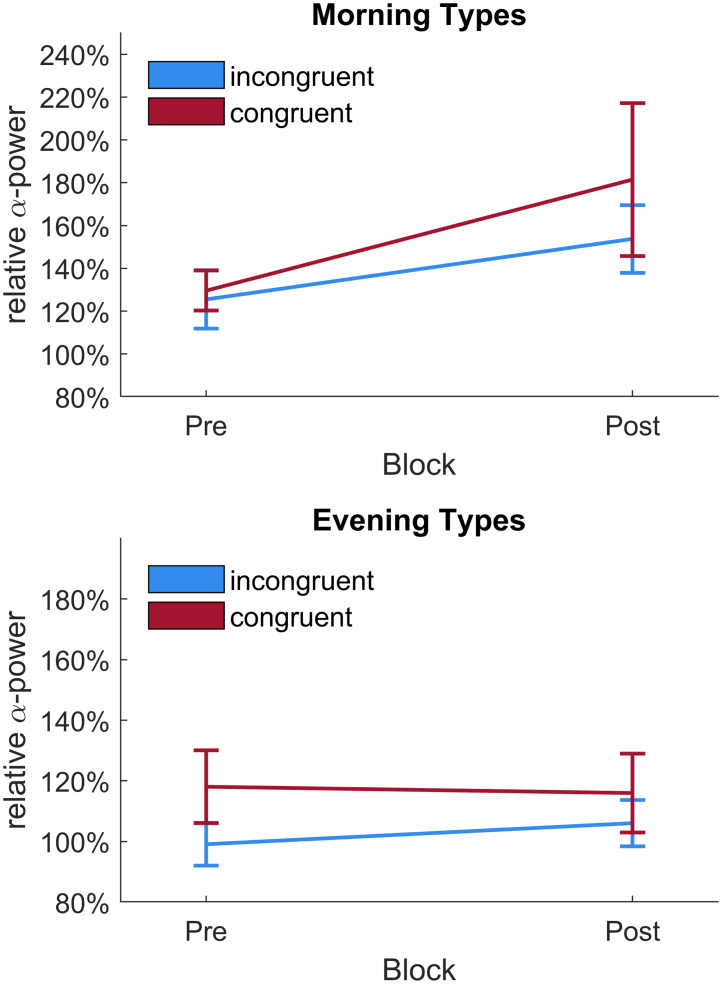
Average normalized power at peak IAF from pre-stimulation to post-stimulation and session type split per chronotype group. Power is normalized to first 5 min of the 10 min pre-stimulation block.

**Fig. 7:**
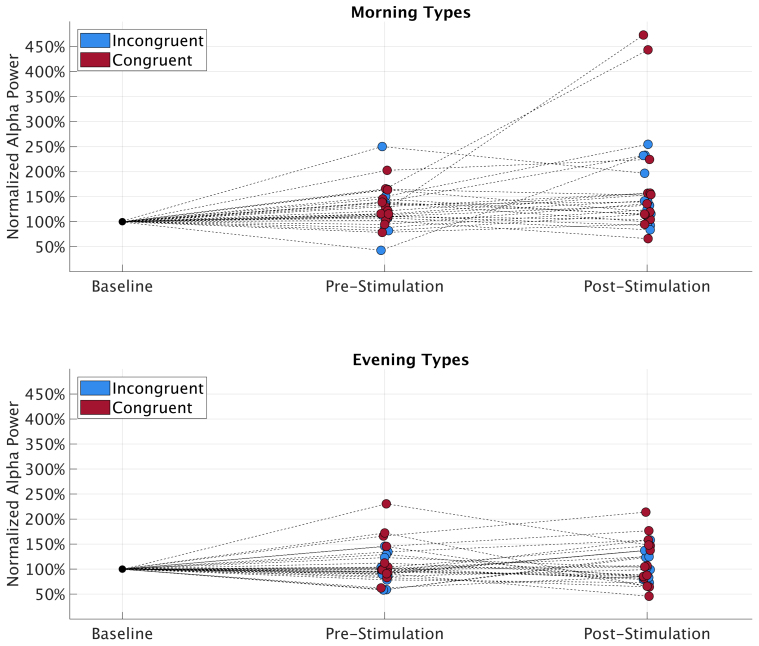
Individual development of normalized alpha power over time split per chronotype and session type. Each dot represents an individual participant’s power at peak IAF averaged per block. Power is normalized to first 5 min in the pre-stimulation block.

Normalized peak power at IAF was entered into a 2 × 2 repeated measures ANOVA with factors time of day x chronotype congruency (congruent vs. incongruent) and block (pre vs. post). Neither congruency (*F*_1,26_ = 1.80, *p* = .19, $\eta_{p}^{2}$ = 0.06) nor block (*F*_1,26_ = 2.24, *p* = .15, $\eta_{p}^{2}$ = 0.08). showed any significant main effect. The interaction between congruency and block (*F*_1,26_ = 0.31, *p* = .58, $\eta_{p}^{2}$ = 0.01) also did not significantly predict peak individual alpha power. For a depiction of average normalized IAF power per Condition and block see Fig. 5. Fig. 6 depicts this split per chronotype. Furthermore, to show the large inter-subject variability, Fig. 7 displays each individual’s normalized alpha power over time. Power spectra of average, non-normalized, power at parietal electrodes are depicted in Fig. 4. An overview of all relative α-power values can be found in Table 2, Table 3.

**Table 2:** Mean normalized power split per condition both chronotypes together.

Condition	mean	sd
con pre	1.2363	0.39514
con post	1.4748	0.99776
inc pre	1.118	0.40574
inc post	1.29	0.50011

**Table 3:** Mean normalized power split per condition and chronotype.

Condition	Type	mean	sd
con pre	ET	1.1806	0.44844
con post	ET	1.1595	0.48639
inc pre	ET	0.99122	0.26326
inc post	ET	1.0607	0.28466
con pre	MT	1.2962	0.33604
con post	MT	1.8144	1.2883
inc pre	MT	1.2545	0.49262
inc post	MT	1.537	0.57155

### 3.4 Exploratory analysis

To check for potential differences between both chronotype groups, it was added to the repeated measures ANOVA as a binary factor. This revealed a significant main effect of chronotype ($F{}_{\text{1,25}}=7.86,\;p=.01$, $\eta_{p}^{2}$ = 0.24). However, there were no interactions of chronotype with the factors block or condition or any changes in their main effects. This suggests the presence of some difference in electrophyisiological outcomes during the vigilance task between both chronotype groups that is not directly explained by the block or condition factors, but rather explained by an unexpected third variable. A Bonferroni-corrected post hoc-test comparing pre-stimulation and post-stimulation alpha power split per chronotype revealed significantly stronger increases in alpha power of morning types compared to evening types in the post-stimulation block (*M*_diff_ = 56.56%, *t*_26_ = 2.30, *p*_corr_ = .03, *d*_av_ = 0.44 (small effect)), but not in the pre-stimulation block (*M*_diff_ = 18.95%, *t*_26_ = 1.73, *p*_corr_ = .10, *d*_av_ = 0.33 (small effect)). Similarly, another Bonferroni-corrected post hoc-test comparing normalized alpha power between congruent and incongruent sessions split per chronotype revealed significantly stronger increases in alpha power of morning types compared to evening types in the incongruent session (*M*_diff_ = 36.98%, *t*_26_ = 3.07, *p*_corr_ = .01, *d*_av_ = 0.59 (medium effect)). In the congruent session this difference was not significant (*M*_diff_ = 38.53%, *t*_26_ = 1.75 *p*_corr_ = .09, *d*_av_ = 0.33 (small effect)).

#### 3.4.1 Baseline difference

Neuling et al. (2013) emphasized the importance of pre-stimulation baseline alpha power and suggested, that with high baseline alpha power, ceiling effects can appear that can interfere with stimulation effects. In order to check for such baseline alpha activity effects and whether they might appear differently between conditions, we compared both frequency and power in the first 5 min of the pre-stimulation block across conditions. As power was not normally distributed, a Wilcoxon signed-rank test was performed. No significant difference was found in baseline power across conditions (W=213,z=0.58,p=0.56). Individual alpha frequency showed an approximate normal distribution. A paired-sample t-test showed no difference between conditions during the baseline period ($t{}_{\text{26}}=-1.20,p=.24$).

#### 3.4.2 Individual alpha frequency

Previous studies have reported a mismatch between the stimulation frequency and IAF as a potential factor for the efficacy of tACS (Kasten et al., 2019, Stecher and Herrmann, 2018). In contrast, some studies did not find significant effects of a potential mismatch (Vossen et al., 2015, Steinmann et al., 2022). The stimulation frequency as determined in the 3 min resting state recording showed a significant, but weak difference between the congruent and incongruent session (*t*_26_ = 2.32, *p* = .03, *d*_av_ = 0.20). On average, stimulation frequency was 0.24 Hz higher in the congruent (*M* = 10.37, *SD* = 1.14) compared to the incongruent session (*M* = 10.13, *SD* = 1.08). In the congruent session, the difference between the stimulation frequency and the mean IAF over all three blocks showed a small but significant difference in a paired-sample t-test (*M*_diff_ = 0.54, *SD*_diff_ = 0.52, *t*_26_ = 3.35, *p* = .003, *d*_av_ = 0.35 (small effect)). This difference was not significant in the incongruent session (*M*_diff_ = 0.55, *SD*_diff_ = 0.49, *t*_26_ = 1.90, *p* = .07, *d*_av_ = 0.22 (small effect)).

#### 3.4.3 Influence of sleep quality

**Fig. 8:**
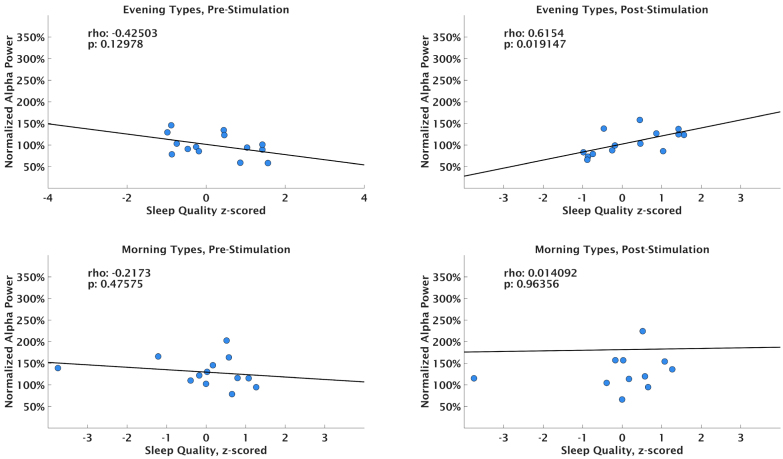
Scatter plot of normalized sleep quality plotted against normalized alpha power in the morning sessions, split per block and chronotype. Each circle represents a single subject.

As we uncovered a potential group difference between chronotype groups, we exploratively decided to check for potential differences in sleep parameters captured by the sleep questionnaire and whether these might explain differences in electrophysiological outcomes. While there was no relationship on the group level between sleep quality and normalized posterior alpha power after stimulation (*p* = .96), splitting the data based on chronotype and time of day revealed a correlation of normalized sleep quality with posterior alpha power after stimulation in the morning condition for evening types only (*r* = 0.62, p = .02) but not for morning types (all *p* > .48), see Fig. 8. No such relationship was observed in the evening condition. Interestingly, normalized sleep time showed no such correlations with alpha power (all *p* > .43). Importantly, as these results stem from exploratory post-hoc subgroup analyses, their statistical power is greatly reduced compared to our main analysis and should therefor not be taken as evidence for the existence of a true effect without further systematic, sufficiently powered studies.

## 4 Discussion

The present study investigated potential differences in aftereffects of tACS in the alpha band due to chronotype and circadian phase mismatches. We expected significantly higher alpha power at a chronotypically preferred time. However, no significant differences in average normalized amplitude after stimulation at peak IAF between the chronotype congruent and incongruent sessions were observed.

Furthermore, a general increase in alpha power after stimulation was expected but not found. Contrary to our expectations, normalized alpha power after stimulation showed quite high variability, with a subgroup that showed decreasing alpha power.

Resting state alpha frequency measured in the 3 min resting state period was significantly higher at the optimal time of day. However, this difference was not observable during the vigilance task.

Lastly it was hypothesized that sleep time and quality alone would not account for the expected differences in alpha power change between sessions. No conclusive answer is possible for this hypothesis, as we did not find any differences in the main analysis. While the present data cannot give a conclusive answer for the effects of chronotype and sleep in α-tACS, exploratory analyses revealed some differences in electrophysiological outcomes between both chronotype groups. This led us to further exploratory analyses regarding the effects of sleep, which hinted at potential negative effects of poor sleep in the previous night.

In the present study, evening types slept significantly fewer hours before the morning session compared to morning types as well as compared to the evening session. While there were no group-level differences in average sleep quality, visual inspection of the individual data hinted at a subgroup of evening types with below average sleep quality in the morning, whereas all morning types reported average or better sleep. We assume that this reflects that only evening types in the morning session had to adjust their sleeping schedule forward to be able to participate and thereby creating a misalignment between their schedule and their circadian rhythm. Such circadian misalignments can increase sleepiness (Chellappa et al., 2019) and could have introduced social jetlag like symptoms, which can lead to worsened sleep quality and resulting difficulties concentrating and daytime fatigue (Roenneberg et al., 2019).

Negative associations between self-rated sleepiness and the magnitude of tACS aftereffects have been reported previously (Steinmann et al., 2022). While sleepiness was not measured directly in the present study, sleep quality is negatively correlated with sleepiness (Nelson et al., 2022). It might be that those evening types with short-term social jetlag symptoms in the morning session resulting in potentially higher sleepiness could not show the expected aftereffects, fitting the observed significant, negative correlations between sleep quality and post stimulation alpha power in the morning for evening types. In the late session and for morning types this effect was not observable in the subjective sleep quality ratings. However, an effect of sleepiness might have also been present in the evening session, and especially so for morning types (Facer-Childs et al., 2018), potentially explaining more of the variability in the present study, but could not be captured by the sleep questionnaire.

Thus, while our data does not show any direct relationships between chronotype and tACS aftereffects, it provides preliminary evidence for the existence of modulatory effects of sleep quality, short term social jetlag and increased sleepiness at non-optimal times on both alpha power and tACS aftereffects. However, systematic studies are required to fully explain this relationship. Still, some findings have been reported that suggest such effects:

In general, sleep deprivation and high subjective sleepiness have been associated with changes in cognitive functioning, oscillatory correlates and functional connectivity (Zhang et al., 2024, Wu et al., 2021, Csipo et al., 2021). Some evidence suggests negative effects of social jet lag, similar to those of sleepiness, in cognitive domains (Taillard et al., 2021) and in functional connectivity (Yang et al., 2023). Furthermore, there is evidence to suggest a disruption of neuroplastic changes after sleep loss (Gorgoni et al., 2013), which could impede aftereffects of NIBS. When repeating their previously described study on chronotype effects in TMS and tDCS on people after a night of full sleep deprivation, Salehinejad et al. (2022) found unexpected null-results and reversals of expected effects, suggesting strong interferences of high sleepiness with NIBS effects. Interestingly, these effects showed high inter-individual variance, especially in the sleep deprivation condition, comparable to the high variance in the present study, likely reflecting individual differences in resilience and adaptability to drastic changes in one’s sleep schedule (Weiss and Donlea, 2022). This resilience might be especially low in people with more extreme chronotypes, considering that extreme chronotypes are characterized by difficulties in adapting to changing sleep schedules (Nowack and van der Meer, 2018, Martin et al., 2015).

Generally, NIBS-studies show high inter-individual variability (Ziemann and Siebner, 2015). Recently, the underlying brain state before and during stimulation has been suggested as one important explanatory factor (Bradley et al., 2022). This state dependency of tACS effects has been seen in rapid changing brain states reflecting activity of specific networks (Kasten and Herrmann, 2022), cognitive demand (Violante et al., 2017) or task engagement (Feurra et al., 2019) and longer lasting brain state differences reflecting network activity differences between sessions (Ziemann and Siebner, 2015) or baseline performance differences (Miyaguchi et al., 2018). Sleepiness, especially in experiments focusing on alpha-activity, might reflect a non-optimal brain state for stimulation, as sleepiness has been associated with systematic changes in default mode network activity involving the modulation of alpha activity (Pomares et al., 2019). Alpha-tACS shows comparable systematic fMRI changes (Clancy et al., 2022), suggesting that sleepiness and alpha-tACS compete and potentially mask their effects, if not controlled for (Guerra et al., 2020). The present study adds to this, that special attention should be given to people who might experience above average sleepiness levels during the stimulation, such as people with extreme chronotypes at non-optimal times and, especially so, evening types in the morning (Facer-Childs et al., 2018).

### 4.1 Methodological considerations and limitations

The decision to not include a sham condition was based on the expectation of a potentially stronger effect of the session timing and an underestimation of the intra-subject variance. However, the lack of a sham group made it impossible to disentangle time on task effects and the effects of stimulation on peak IAF power, and whether these effects are modulated differently by sleep quality and related sleepiness. The closest comparison we can draw is with the results of Kasten et al. (2016), who employed the most similar paradigm (same stimulation duration, same stimulation frequency (IAF), same electrode size and sites and same behavioral task). Three to nine minutes after stimulation they found an increase of ∼ 37% alpha-band power in the stimulation condition and ∼ 13.5% in the sham condition. Across chronotypes, our results show an increase comparable to their stimulation condition as can be seen in Table 2. When de-pooling our results into both chronotypes, we see that the increase is mainly carried by the morning types, while the evening types are closer to the sham-results of Kasten et al. (2016). These results could hint at an overall weaker effect of stimulation in evening types, possibly driven by increased sleepiness. However, Kasten et al. (2016) employed only a 3-minutes pre-stimulation block, which offsets their timeline by at least 7 min compared to ours. Additionally, they employed a perceptual-threshold based amplitude for tACS (on average 1.2 mA compared to our 2 mA), so a direct comparison should be interpreted with caution.

Considering the aforementioned time-on-task and sleepiness effects on alpha power, the preparation time must be taken into account. As this is an often under-reported background variable, no accurate comparisons between the preparation time in this study with a typical time can be made. However, on a general note, long preparation times could induce sleepiness when participants feel bored (Danckert et al., 2018). This sleepiness could have increased endogenous alpha activity before stimulation and interfered with aftereffects of stimulation. As absolute power values in the EEG show strong inter-individual variability (Angulo-Ruiz et al., 2021), it is difficult to determine how strongly alpha activity increased until the first EEG measurement. In a comparison between an eyes-open (low baseline alpha) and eyes-closed conditions (high baseline alpha), Neuling et al. (2013) found tACS aftereffects only when baseline alpha activity was low. In a similar vein, the potentially heightened baseline alpha from a combination of the long preparation time and participants’ sleepiness might have led to such ceiling effects, which might explain the comparatively small total power increase and might have masked the influence of chronotype.

The large variance observed in sleep time between sessions and individuals hint towards a potential non-optimal timing of the sessions for at least some participants. Some participants unexpectedly reported worse and shortened sleep before their optimal session compared to their non-optimal one. Additionally, for some morning types, their optimal session might have taken so long, that the stimulation happened outside of this optimal window. Thus, in some cases, circadian phase shifts and mismatches might have been present in both sessions. Considering the established negative effects of these phase shifts and circadian disruptions on cognitive performance such as alertness, attention and inhibitory processes (Taillard et al., 2021), which are at least partly associated with posterior alpha (Klink et al., 2020), it is likely that this presents another confounding variable. Future studies investigating the effects of chronotype on tACS, might benefit from more individualized session-timing to better capture optimal and non-optimal times. Using objective measures of typical patterns of rest and activity, such as sleep diaries or actigraphy (Acker et al., 2021) could increase the accuracy of individualized session timing even further. Alternatively, with fixed timings, these measures could be introduced as covariates. Additionally, the use of such measures would strengthen the evidence for potential chronotype effects by enabling further validation of a person’s subjective estimate for their chronotype. It has been suggested, that some people tend to overestimate their chronotype, especially at extreme ends, when compared to more objective measures (Schneider et al., 2022).

One major limitation for the interpretation of our results for the role of sleep quality is the reliance on post-hoc subgroup analyses (evening types in the morning) which lead to significantly lower statistical power and no within-subject comparison. Ideally, the relationship between sleep quality and normalized alpha power should be compared across chronotype groups and between stim and sham conditions. As we opted not to include a sham group and as the morning chronotype group did not report sleep quality ratings below average, these comparisons and subsequent statistical modeling (e.g. using linear mixed models) were not possible. Therefor our findings should only be interpreted as exploratory and need to be validated in a systematic sham-controlled study in which both groups have at least some observations of below average sleep quality.

We assumed that the effects of sleep quality observed here indirectly reflect sleepiness, due to pre-established links between poor sleep quality and increased daytime sleepiness (Nelson et al., 2022), as well as negative relations shown between sleepiness or sleep disruption and NIBS efficacy (Steinmann et al., 2022, Salehinejad et al., 2022). However, people differ in their resilience to negative effects of poor sleep (Rupp et al., 2012, Poon et al., 2024). Furthermore, there are several other factors that can increase sleepiness, which can vary greatly over the course of a single day and across people (see Hershner and Chervin, 2014 for a review of these factors in college students). While it is reasonable to assume that in the morning session, especially for evening types who generally reported that they came to the lab directly after waking up, excessive sleepiness was mostly caused by poor sleep quality, this relationship was likely less strong in the afternoon session or in those morning types who were active already before the morning session. More direct measures of concurrent sleepiness would be required to accurately describe the relationship between chronotype, time of day, sleep quality, sleepiness and tACS aftereffects.

Reaction times and accuracy in a vigilance task can be used as an indirect measure of sleepiness (Thomann et al., 2014). However, as several of our participants achieved (near) perfect accuracy, it is likely that our task was too easy to be used as an accurate measure of sleepiness. Future studies should employ more difficult vigilance tasks and direct measures of sleepiness to quantify participant’s state during the sessions (see Martin et al., 2023 for an overview of sleepiness measures).

### 4.2 Conclusion and outlook

Despite failing to find the expected direct evidence for an interaction between chronotype misalignment and the strength and presence of tACS aftereffects, the present study provides preliminary evidence that low sleep quality and associated sleepiness might weaken tACS aftereffects at chronotypically non-optimal times, falsely suggesting the absence of such aftereffects if sleep is not controlled for.

Furthermore, it emphasizes the importance of individual variability, even in within-subject designs. Some participants unexpectedly showed no increases or even decreases in alpha power after stimulation, which could partly be explained by below average sleep quality and resulting high social jet lag-like symptoms (e.g. increased sleepiness) for evening types before the morning session. Generally, sleepiness seems to weaken tACS aftereffects (Steinmann et al., 2022). Circadian misalignment can increase sleepiness and fatigue (Chellappa et al., 2019). Thus, we recommend that appropriate measures are taken to control for sleep quality and sleepiness, with special attention paid to situations in which circadian misalignments are most likely either in measurements taking place early in the morning or late in the evening or in participants with strong chronotypes. As our design only allowed for an indirect measure of sleepiness at one time point, future studies on chronotype effects in NIBS should systematically investigate not just sleep quality and time but also sleepiness and fatigue. Ideally a full within-subject sham vs. stim design comparing both morning and evening types at optimal versus non-optimal times, including measures of sleep quality before and subjective sleepiness during the session, should be implemented (Vogeti et al., 2022). Such a full within-subject design with a larger sample size would allow for modeling inter-subject variability using random effects as well as for modeling time-varying effects of sleep quality and sleepiness using linear mixed models to potentially shed more light on the role of chronotype and time of day in tACS. Several recent studies employed models that account for similar covariates and were able to describe effects which would not be observable using simple ANOVA methods (Antonenko et al., 2016, Steinmann et al., 2022, Stecher and Herrmann, 2018).

Lastly, the present study highlighted the importance of controlling and reporting background variables to enable a better understanding of the variability of effects currently encountered in NIBS and to increase reproducibility (Hensel, 2020). We recommend any study investigating potential chronotype effects in NIBS to assess and properly account for behavioral correlates such as sleep, alertness, task performance and other relevant measures, regardless of the outcome for the main hypotheses. In tACS research this is even more important than in neuroscience in general, considering the growing evidence for a state dependency of tACS effects (Kasten and Herrmann, 2022). Thus it is vital for the whole field of NIBS to identify, measure, report and potentially control for such factors with the ultimate goal of reliable predictions for the efficacy of NIBS on the individual level.

## CRediT authorship contribution statement

**Peppi Schulz:** Writing – original draft, Visualization, Software, Methodology, Investigation, Formal analysis, Data curation, Conceptualization. **Heiko I. Stecher:** Writing – review & editing, Software, Methodology, Formal analysis. **Christoph S. Herrmann:** Writing – review & editing, Supervision, Methodology, Funding acquisition.

## Funding

CSH was funded by the German Research Foundation (Deutsche Forschungsgemeinschaft, DFG) under Germany’s Excellence Strategy (EXC 2177/1 - Project ID 390895286) and the research training group on Neuromodulation of Motor and Cognitive Function in Brain Health and Disease (RTG 2783).

## Declaration of competing interest

The authors declare the following financial interests/personal relationships which may be considered as potential competing interests: Christoph S. Herrmann has patent #11110268 issued to CARL VON OSSIETZKY UNIVERSITÄT OLDENBURG. If there are other authors, they declare that they have no known competing financial interests or personal relationships that could have appeared to influence the work reported in this paper.

## Data Availability

Data and Code is made available at https://osf.io/u3w2t/?view_only=36d9a928741945738e9396c9b35cc38f.
